# The Gut Microbiota and Vascular Aging: A State-of-the-Art and Systematic Review of the Literature

**DOI:** 10.3390/jcm11123557

**Published:** 2022-06-20

**Authors:** Davide Agnoletti, Federica Piani, Arrigo F. G. Cicero, Claudio Borghi

**Affiliations:** 1 Cardiovascular Internal Medicine, IRCCS Azienda Ospedaliero-Universitaria di Bologna, 40138 Bologna, Italy; federica.piani2@unibo.it (F.P.); arrigo.cicero@unibo.it (A.F.G.C.); claudio.borghi@unibo.it (C.B.); 2 Cardiovascular Internal Medicine, Medical and Surgical Sciences Department, University of Bologna, 40138 Bologna, Italy

**Keywords:** vascular ageing, arterial stiffness, central hemodynamics, pulse wave velocity, gut microbiota, gut microbiome, inflammation, oxidative stress

## Abstract

The gut microbiota is a critical regulator of human physiology, deleterious changes to its composition and function (dysbiosis) have been linked to the development and progression of cardiovascular diseases. Vascular ageing (VA) is a process of progressive stiffening of the arterial tree associated with arterial wall remodeling, which can precede hypertension and organ damage, and is associated with cardiovascular risk. Arterial stiffness has become the preferred marker of VA. In our systematic review, we found an association between gut microbiota composition and arterial stiffness, with two patterns, in most animal and human studies: a direct correlation between arterial stiffness and abundances of bacteria associated with altered gut permeability and inflammation; an inverse relationship between arterial stiffness, microbiota diversity, and abundances of bacteria associated with most fit microbiota composition. Interventional studies were able to show a stable link between microbiota modification and arterial stiffness only in animals. None of the human interventional trials was able to demonstrate this relationship, and very few adjusted the analyses for determinants of arterial stiffness. We observed a lack of large randomized interventional trials in humans that test the role of gut microbiota modifications on arterial stiffness, and take into account BP and hemodynamic alterations.

## 1. Introduction

For several decades, cardiovascular disease (CVD) has been the leading cause of death worldwide. CVD is mainly driven by high blood pressure (BP), which causes damage several target organs. However, there is some evidence that cardiovascular risk due to hypertension is not fully restored by antihypertensive treatment, leading to the concept of residual cardiovascular risk [1]. This is in line with the hypothesis that underlying factors drive both CVD and hypertension, and precede the clinical evidence of the disease, even before hypertension is established. Indeed, subclinical local and systemic inflammation could be one of the main drivers of target organ damage. One of the underlying factors contributing to increased cardiovascular risk is arteriosclerosis, a process of progressive stiffening of the arterial tree associated with arterial wall remodeling, which can precede hypertension and organ damage during the life course. This process has recently been mentioned as “vascular ageing” [2].

The main function of the arterial system is to dampen the pulsatility induced by the stroke volume during the systole.

This is of pivotal importance as organs with high flow and low resistance (e.g., the heart, brain, kidney) are prone to the side effects of increased pulsatility [3]. The large arteries, mainly the aorta, due to their elastic properties, contribute to preserving continuous and low pulsatile flow into target organs. The progressive stiffening of the arterial tree from the center to the periphery, together with the phenomenon of pressure wave reflection, maintains and even amplifies blood pressure in order to guarantee correct organ perfusion. Eventually, the peripheral resistances preserve the target organ microcirculation from pulsatility. During the physiological process of ageing, the arterial tree becomes more and more stiff, due to a change in the elastin–collagen ratio and to the deterioration of the arterial extracellular matrix. This leads to increased pulsatility at the peripheral level, where target organs may be injured. Early vascular ageing (EVA) is an attempt to describe early vascular modifications, leading to a stiffer arterial tree as compared with the normal aging process, and conferring higher cardiovascular risk. Arterial stiffness has become the preferred marker of EVA and is easily estimated by the measurement of pulse wave velocity (PWV). The stiffness of an arterial segment can be estimated either “locally” (e.g., in the carotid artery by the doppler ultrasound technique, or in the ascending aorta by magnetic resonance) or “globally” (e.g., the entire aorta by the carotid-femoral PWV [cfPWV] by arterial tonometry). Aortic PWV has become the gold standard for the estimation of aortic stiffness and is an established marker of cardiovascular morbidity and mortality [4,5].

The gut microbiota has emerged as a critical regulator of human physiology, and deleterious changes of its composition and function, commonly referred to as dysbiosis, have been linked to the development and progression of numerous disorders, including cardiovascular diseases. In particular, both direct and indirect roles of gut microbiota have been described on blood pressure regulation and vascular inflammation and stiffening.

The human gastrointestinal tract harbors a vast array of microorganisms that significantly affect host nutrition, metabolic function, gut development, and maturation of the immune system and intestinal epithelial cells [6,7,8]. In the present review, we refer to “microbiota” as the composition of the whole gut bacterial microorganism. Overall, the microbiota comprises 5 major phyla and approximately more than 1000 species in the large intestine. The gut microbiota promotes digestion and food absorption for host energy production, whereas in the colon, complex carbohydrates are digested and subsequently fermented into short chain fatty acids (SCFAs) such as n-butyrate, acetate, and propionate. The resulting SCFAs seem to regulate neutrophil function and migration, reduce colonic mucosal permeability, inhibit inflammatory cytokines, and control the redox environment in the cell. From a physiological point of view, the main producers of SCFAs belong to the Firmicutes phylum, the single largest grouping of gut bacteria. The Clostridia class in the Firmicutes phylum includes diverse bacteria of medical, environmental, and biotechnological importance. In particular, butyrate and butyrate-producing microbes have been associated with gastrointestinal health in humans and various animal species, and in the human gut are predominately members of clostridial clusters IV (phylum Firmicutes, class Clostridia, genera: *Faecalibacterium*, *Oscillibacter*, *Ruminococcus*, …) and XIVa (phylum Firmicutes, class Clostridia, genera: *Coprococcus*, *Roseburia*, *Clostridum_g24*, …) [9]. *Clostridium* clusters XIVa and IV represent the gut’s predominant bacteria, accounting for 10–40% of the total bacteria [10]. *Akkermansia muciniphila*, the only member of the Verrucomicrobia phylum, is one of the SCFA-producing bacteria and represents 3–5% of total faecal microbes. A large number of studies has shown that the abundance of *Akkermansia* in the gut is correlated with several health benefits in humans [11]. These beneficial effects are related to the ability of the bacterium of maintaining the mucus thickness and the integrity of the intestinal barrier, providing energy sources (SCFAs) for mucin-producing goblet cells [11]. Studies have shown a relationship between low *A. muciniphila* abundance and increased occurrence of inflammatory metabolic diseases, such as diabetes, obesity, and inflammatory bowel disease [12], which are associated with epithelial gut damage and high permeability.

In investigating gut microbiota characteristics, it is important to explore (i) α diversity: the microbial diversity at the smallest spatial scale (intra individual), assessed by the Shannon or Simplon index; (ii) ß diversity: the microbial diversity at the landscape scale (inter-individual diversity within the same population); (iii) Richness: the total number of species in the unit of study, measured as operational taxonomic units (Chao1 index); (iv) Firmicutes:Bacteroidetes (F:B) ratio: an index of balance between the two most relevant bacterial families. Furthermore, a high-resolution analysis of the bacterial community down to the species level, and a functional profiling for the assessment of the most represented genes/metabolic pathways and their relative abundance is obtained by a metagenomics analysis, whereas metatranscriptomics is exploited to elucidate which microbial genes annotated in metagenomes are actually transcribed and to what extent. The abundance of health-promoting or detrimental microbiome-derived metabolites (e.g., lipidome and metabolome profile) is assessed by metabolomics analysis.

**Figure 1:**
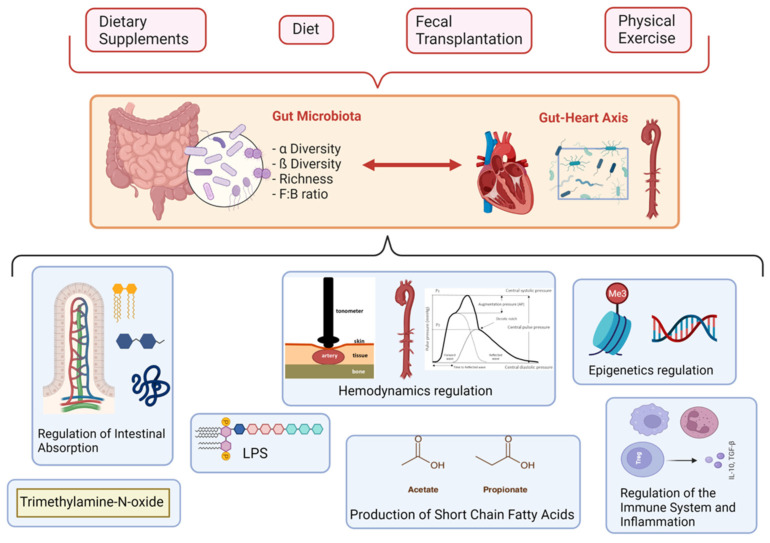
Schemes of the intercorrelation between environmental and biological mechanisms, gut microbiota, and vascular ageing.

The mechanisms underlying the association between vascular ageing and the gut microbiota are presented in Figure 1.

## 2. State-of-the-Art Review

### 2.1. Microbiota and Hypertension

Data from literature indicate that fruit and vegetable intake is associated with both lower BP values and reduced cardiovascular mortality [13,14], despite a high fat intake [15]. Several micronutrients have been investigated as potential proactive means to achieve such results. Fibers interact with gut microbiota, stimulating growth of specific bacterial phyla, and fiber intake has been associated with lower cardiovascular and all-cause mortality [16].

Among the metabolites produced by the gut microbiota, alanine, n-methylnicotinate, hippurate, and formate have been associated with BP levels. While formate and hippurate are negatively correlated with BP, alanine (produced mainly under a carnivore diet) is associated with higher BP [17]. 4-hydroxyhippurate production by polyphenols microbial metabolism was associated with higher risk of developing hypertension at 10 years (1.17 [95%CL 1.08–1.28]) in a black normotensive population [18]. Subjects with prehypertension or stage 1 hypertension underwent a randomized crossover trial with three diet interventions: carbohydrate-rich, protein-rich, or mono-unsaturated fat-rich diet for 6 weeks each. Urinary metabolites were associated with blood pressure, in particular: proline-betaine (derived from carbohydrate, protein and fat-rich diets), 4-cresyl sulfate and phenylacetylglutamine (derived from the fat-rich diet), N-methyl-2-pyridone-5-carboxamide (derived from the carbohydrate-rich diet) were inversely associated with BP, while carnitine (derived from the protein-rich diet) and hippurate (derived from the carbohydrate-rich diet) were positively associated with BP levels [19]. These results show that different diet interventions, addressing macronutrient content, are associated with distinct hemodynamic effects.

From the available data, hypertension is associated with gut microbiota dysbiosis, characterized by an increased F:B ratio, as well as a drastic decrease in acetate-, butyrate-, and an accumulation of lactate-producing microbial populations. Treatment with an oral minocycline dose, which interferes with microbial growth, has been found to attenuate hypertension and produce beneficial effects on dysbiosis in a rat model [20]. Otherwise, primary alterations in the gut microbiota may elicit hypertension, as was highlighted by an animal study where hypertensive, stroke-prone rats (SHRSP) presented a dysbiotic gut. The main result was that, after transplantation of the SHRSP microbiota in normotensive rats, the authors observed a significant increase in systolic blood pressure (SBP) [21].

If the relationship between gut microbiota and hypertension is so far well described, it is not easy to understand by which pathophysiological mechanisms do the microbial environment and its metabolites regulate BP levels. One of the main characters in the scene is the group of SCFAs.

### 2.2. The Role of SCFAs

For more than two decades, SCFAs, mainly acetate, propionate and butyrate, have been found to be involved in dilatation in rat tail arteries and human colonic resistance arteries in a concentration-dependent way [22,23,24]. More recent findings suggest that propionate enhances renin release from juxta glomerular cells, and reduces BP levels in hypertensive mice, as well as in wild mice [25]. In a model of deoxycorticosterone acetate (DOCA)-salt mice, chronic acetate intake was associated with lower BP levels, together with reduced myocardial fibrosis and hypertrophy, and better cardiac function [26]. Interestingly, in the same study, the authors describe that fiber intake could modify the gut microbiota, increasing acetate-producing bacteria, and that fiber and acetate supplementation improved dysbiosis. The positive biological effects of fibers and acetate were ascribed to: (i) the downregulation of cardiac and renal genes for early-growth-response-protein-1 (Egr1), involved in myocardial hypertrophy, fibrosis, and inflammation; (ii) the downregulation of the renin–angiotensin system in the kidney [26].

Several metabolite-sensing G-protein-coupled receptors (GPCRs) have been found to bind with SCFAs, and are important for gut health and immune response regulation [27]. Impaired signaling of these receptors may occur due to excess fat or sugar intake, and could be involved in the deterioration of the intestinal barrier, with lipopolysaccharide (LPS) translocation and subsequent local and systemic inflammation (leaky gut syndrome, see paragraph 2.6) [27,28]. In addition, SCFAs could influence cellular gene expression, binding to diverse histone deacetylases. One of the most important GPCRs is the olfactory receptor 51E2 (named Olfr78 or OR51E2), which is found mainly in arterial smooth muscle cells, autonomic nerves, and in juxtaglomerular apparatus, and binds acetate and propionate, resulting in increased renin release. In Olfr78-/- mice, both BP and plasma renin levels were found to be lower than in wild mice. Indeed, a challenge of propionate in wild mice resulted in a dose-dependent BP lowering, while in Olfr78-/- mice, an accentuated BP-lowering effect was observed with a very low propionate dose, indicating that Olfr78 activation antagonizes the acute hypotensive effect of propionate. This suggests that propionate receptors other than Olfr78 regulate BP levels [25]. GPR41 is found in the vascular endothelium and mediates vasodilation through SCFA stimulation. GPR41-/- mice are not prone to the BP-lowering effect of propionate, and present both higher BP levels and arterial stiffness [29]. This realizes a complex schema where the same metabolite (propionate) can increase renin release, but can also exert a hypotensive effect, depending on the receptor it activates.

According to the data presented, it appears that a biological link exists between microbiota composition, the production of SCFAs, and blood pressure regulation. In particular, beside the effect of blood pressure, it is worth highlighting that SCFA production is almost constantly associated with a beneficial microbiota composition, and that higher butyrate levels are mostly associated with positive local and systemic effects. They include: improvement of colonocytes health; reduction of neutrophils migration; increased tight junction protein, and an anti-inflammatory effect.

### 2.3. Maternal Heritage and Genetic/Epigenetic Regulation

A study on mice fed with a high-fiber diet or acetate during pregnancy showed that acetate inhibited the histone deacetylaese-9, which resulted in the downregulation of atrial natriuretic peptide (ANP) in the offspring [30]. In another mouse model, mice fed with fiber or acetate presented an improved heart and kidney function, in particular through genetic regulation of fibrosis, fluid absorption, the renin angiotensin system, and inflammation pathways (e.g., by downregulation of transcription factor Egr1, IL-1, Rasal1, Cyp4a14, Cck) [26].

In offspring exposed in utero to maternal obesity, a mouse study found a specific methylation profile in the resistance of mesenteric arteries, which was associated with vascular remodeling and impaired vasodilation [31].

Overall, from the scarce information available, it seems that maternal characteristics and behavior influence offspring in terms of both inflammation pathways and hemodynamics.

### 2.4. Inflammation and Immune System in Hypertension

Hypertension is associated with immune activation. Increased numbers of central memory CD8+ T cells, activated CD8+ T cells producing Interferon-gamma (IFNγ) and tumor necrosis factor (TNF), and TH17 cells have been reported in patients with hypertension [32]. Monocytes from patients with essential hypertension are preactivated, producing greater amounts of IL-1β, TNF and IL-6 following ex vivo stimulation with angiotensin II or LPS than monocytes from healthy controls [33,34]. A major goal of hypertension treatment is the prevention of end-organ damage. A substantial portion of the vascular, renal, cardiac, and brain damage and dysfunction that accompanies hypertension is mediated by inflammation within these target organs. The innate and adaptive immune responses are critical to the development of hypertension and its consequences [35]. Adaptive immunity activation of both T-cells and B-cells is initiated early in the course of the disease and greatly contributes to important pathogenetic changes, through release of pro-inflammatory cytokines and antibodies [34].

While hypertension and aging are established factors contributing to arterial stiffness, the role of inflammation in the stiffening of the arteries is less well understood.

Arterial stiffness is associated with increased production of reactive oxygen species [36] and proinflammatory cytokines [37]. Furthermore, C-reactive protein itself may play an active role in mediating arterial stiffening, by inducing endothelial dysfunction. The increased vascular inflammation increases vascular fibrosis, smooth muscle cell proliferation, and impair endothelial-mediated vasodilation, which subsequently leads to increased arterial stiffness [38]. Oxidative stress appears to play a role in the pathogenesis of arterial stiffness, as oxidative injury may result in increased vascular inflammation and increased cellular proliferation, which may subsequently lead to impaired arterial elasticity [39].

Multiple studies have shown elevated indices of arterial stiffness in subjects with primary inflammatory disorders, and prospective studies (including 2 RCTs) have demonstrated a reduction in arterial stiffness following treatment with anti-TNF and other anti-inflammatory agents [40,41,42,43,44].

The gut microbiota is thought to modulate immune and inflammatory responses. Germ-free mice present lower levels of TH7 and Treg, and a higher TH2/TH1 ratio than wild mice, which is associated with hypertension development. Furthermore, GPCR are also localized in immunity cells, so that SCFAs are able to interact with and activate them [45]. From the evidence available so far, it is clear that: (i) Inflammatory dysregulation occurs during human hypertension, together with immune system activation; (ii) Vascular ageing, as estimated by arterial stiffness, is strictly influenced by inflammation and oxidative stress, and could precede the development of hypertension; (iii) The gut microbiota can regulate immunity cells and the inflammation response.

### 2.5. The Role of Trimethylamine-N-Oxide

The pivotal role of the gut microbiota in cardiovascular disease is highlighted by the data concerning trimethylamine-N-oxide (TMAO) and its link to both microbiota and atherosclerosis. TMAO is a molecule transformed from the metabolism of choline by the gut microbiota, starting from dietary phosphatidylcholine. The main sources of phosphatidylcholine are meat, eggs, and foods with a high cholesterol content. The final TMAO plasma concentration depends on diet, gut microbial composition, drugs, and the activity of the liver flavin monooxygenase [46,47].

Plasma TMAO concentration correlates with incidents of major adverse cardiovascular events in patients with acute coronary syndrome [48]. TMAO levels were also associated with ageing, systolic blood pressure, and cfPWV, independent of cardiovascular risk factors [49]. TMAO dietary supplementation increased arterial stiffness in both young and old mice, impaired aortic wall intrinsic mechanical stiffness, and increased aortic wall concentration of advanced glycation end-products [49]. Interestingly, in mice, TMAO infusion amplified the angiotensin II effect in increasing BP [17,50], but did not affect BP levels in normotensive rats, so that it is unclear whether TMAO is proatherogenic or a marker of atherosclerosis [50]. In any case, even if the results of an experimental study show an obligate role for intestinal microbiota in the generation of TMAO from the dietary lipid phosphatidylcholine [51], it is still not clear whether specific patterns of microbiota composition would be associated with different levels of TMAO production. This issue is highlighted by the results of a recent meta-analysis showing that supplementation with probiotic *Lactobacillus rhamnosus GG* was the most efficient in reducing the plasma TMAO level in both humans and animals [52].

### 2.6. The Role of Lipopolysaccharides

LPSs are found mainly in the gut lumen, as they form the outer membrane of gram-negative bacteria. In situations of increased permeability of the epithelial gut barrier, LPS transmigrates into the blood stream, and it binds to the toll-like receptor-4 by means of CD14 complex. This stimulation induces the release of several proinflammatory cytokines by the NF-kB pathway, with activation of the immune and inflammatory response [53].

Even if LPS enhances the atherosclerotic process and boosts the formation of unstable plaques, its link with hypertension is still debated.

Gut microbiota dysbiosis easily induces both an increase of lumen LPS and a deterioration of the gut epithelial barrier with amplification of tight junction permeability, resulting in the transmigration of LPS; this phenomenon is called the “leaky gut syndrome”, and is one of the promoters of systemic inflammation driven by the gut microbiota.

### 2.7. The Role of Salt

As shown above, different dietary patterns, including a diverse composition of fiber, fructose, and fat, modulate the gut microbiota with various effects on inflammation, the immune system, and BP levels. In this domain, dietary sodium intake has emerged as an important player for its interaction not only with BP levels, but also with gut microbiota, inflammation, and the immune system [54,55].

Sodium and water absorption are regulated by the sodium–proton exchanger 3 (NHE3), which is found both in the gastrointestinal tract and the renal proximal tubule [56]. In a model of spontaneously hypertensive rats, the inhibition of NHE3 resulted in increased fecal content of sodium and water, decreased urinary sodium excretion, and lower BP levels [57]. NHE3-ko mice presented altered gut microbiota, with a beneficial decrease of the F:B ratio [58], but NHE3 deficiency was also found to induce irritable bowel syndrome, with gut dysbiosis [59], making the results difficult to interpret. High salt intake alters gut microbiota, inducing low microbial diversity [60] and the depletion of *Lactobacillus* spp., which is restored after normalization of the sodium dietary content [61]. Moreover, supplementing *Lactobacillus* reduced BP levels and TH-17 cell activation in mice fed with a high-salt diet [61]. Salt intake also affects the Clostridial order, with a reduction of several genera, and an increase in Christensenellaceae, Corynebacteriaceae, Lachnospiraceae, Ruminococcaceae and *Oscillospira*, with exacerbation of colitis [60,62]. It is worth noting that the biological result of the modifications of the genera abundances in the gut is not always predictable, as it depends also on bacterial species-to-species interaction and on activation of specific genes. Indeed, mice fed with a high-salt diet show a higher abundance of *Roseburia*, a butyrate-producing species [60], but lower butyrate production, perhaps due to the loss of interaction with *Lactobacillus* spp., which is depleted [62].

High salt intake can increase several proinflammatory cytokines, such as interleukin (IL)-6 and IL-23 [63], and may activate TH-17 cells with production of IL-17 and IL-22 [55,61], which are associated with the development of hypertension [64]. Interestingly, as shown before, these activation mechanisms are likely mediated by the gut microbiota [45,65].

From the data presented here, it emerges that salt intake is associated with several biological mechanisms related to disbiosis and inflammatory pathways.

### 2.8. Microbiota and Exercise

A relatively recent observational study comparing the fecal bacterial profile of elite male rugby players with non-athlete healthy subjects [66] showed significant differences between the two groups; in particular, athletes had lower levels of Bacteroidetes and greater amounts of Firmicutes than the controls. After analyzing the gut microbiota composition of the participants of the American Gut Project, it was concluded that increasing exercise frequency from never to daily causes greater diversity among the Firmicutes phylum (including *Faecalibacterium prausnitzii* and species from the genus *Oscillospira*, *Lachnospira*, and *Coprococcus*), which contributes to a healthier gut environment. In the limited studies available in animal models, exercise in rats was associated with higher Bacteroidetes and lower Firmicutes in fecal matter, whereas the cecal microbiota following 6 weeks of exercise activity presented a greater abundance of selected Firmicutes species and a lower abundance of *Bacteroides/Prevotella* genera. Similarly, at the phyla level, exercise reduced Bacteroidetes, while it increased Firmicutes, Proteobacteria, and Actinobacteria in mice. Even if data on bacterial genera are lacking, this microbiota composition could represent a benefical adaptation to exercise. Rats that participated in voluntary running exercise had increased colonic butyrate concentrations compared to sedentary rats, due to higher levels of butyrate-producing bacteria from the Firmicutes phylum (SM7/11 and T2-87) in their cecum. Hsu et al. investigated the influence that intestinal microbiota has on endurance swimming time in specific pathogen-free (SPF), germ-free (GF), and *Bacteroides fragilis* (BF) gnotobiotic mice. They found that the antioxidant capacity was deeply different in the three mice models, as serum levels of glutathione peroxidase (GPx) and catalase (CAT), two major antioxidants able to convert hydrogen peroxyde into water, were greater in SPF than GF mice. Additionally, serum superoxide dismutase (SOD) activity, pivotal for the clearance of superoxyde radicals, was lower in BF than SPF and GF mice. The authors found that endurance swimming time was longer for SPF and BF mice than GF mice, suggesting that the gut microbiota composition is crucial for exercise performance, and could also be linked to the activity of antioxidant enzyme systems. The types and amount of SCFAs produced by gut microorganisms are determined by the composition of the gut microbiota and the metabolic interactions between microbial species, but also by the amount, type, and balance of the main dietary macro- and micronutrients [67].

Exercise training seems to have a role in gut microbiota composition and function, and the bacterial patterns may evolve during exercise, potentially providing beneficial adaptation to physical stress. At the same time, the gut microbiota composition itself may influence the exercise performance.

### 2.9. Nutrition and Stiffness

The relationship between dietary components and arterial stiffness has been investigated in limited and heterogeneous studies that seem to indicate a beneficial effect of certain nutrients on vascular ageing.

Higher anthocyanin and flavone, cocoa intake, as well as phytoestrogens such as isoflavones and lignans, are associated with lower arterial stiffness [68].

Dietary polyphenols have been investigated in several small heterogeneous studies. Cocoa and chocolate, rich in flavonoids and proanthocyanidins, seem to reduce BP levels and cardiovascular risk, with an improvement in measures of vascular health (arterial stiffness and endothelial function), possibly due to the activation of nitric oxide (NO) synthase, and to other antioxidant/anti-inflammatory properties [69,70]. The European Food Safety Authority approved a health claim about the effectiveness of cocoa polyphenols on arterial elasticity, indicating an ideal assumption of 200 mg of cocoa flavanols daily, consumed as 2.5 g high-flavanol cocoa powder, or 10 gr high-flavanol dark chocolate [71]. Although anti-inflammatory and antioxidant effects have been associated with berry and grape juice consumption, there are no sufficient data to establish their relationship with arterial stiffness. On the other hand, the importance of isoflavone (a soy metabolite) in reducing arterial stiffness and BP levels has been highlighted [72].

Curcumin capsule supplementation has shown to reduce PWV in diabetic patients in a randomized trial [73].

## 3. Systematic Review

### 3.1. Aim

This systematic review aims to investigate (i) the interdependence between gut microbiota composition and central hemodynamics, and (ii) whether modifications to gut microbiota translate into different vascular aging profiles.

### 3.2. Methods

#### 3.2.1. Eligibility Criteria

This systematic review is based on population, intervention, comparator, outcome, and setting criteria. Participants: humans or animals included in both observational and interventional studies. Interventions: we considered every kind of intervention (dietary, antibiotics, fecal transplant, dietary supplements, etc.). Comparators: we included any kind of comparator. Outcomes: primary outcomes: (i) modification in PWV; (ii) modification in gut microbiota composition (alpha- and beta-diversity, genera abundances); secondary outcome: relationship between changes in PWV and gut microbiota composition. Study designs: observational, experimental, and interventional trials in humans and animals are included. No restrictions were imposed on language or date of publication. Exclusion criteria: studies without information about either microbiota or arterial stiffness were excluded; editorials, study protocols, reviews, commentary, and letters were also excluded.

#### 3.2.2. Information Sources and Search

The following databases from inception to February 2022 were searched: PubMed/MEDLINE, Scopus, Web of Science. The main electronic search strategy was designed for PubMed/MEDLINE and was adapted as appropriate for each of the other databases.

D.A. and F.P. screened titles, abstracts, and full texts of articles identified in this search, and extracted the data for eligible studies; discrepancies were resolved by consensus.

### 3.3. Results

**Figure 2:**
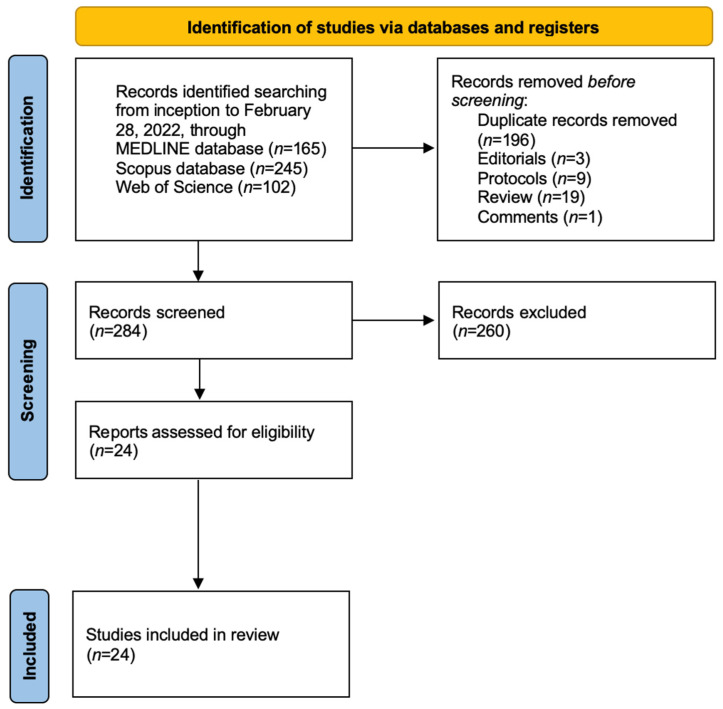
Flow diagram of the Systematic Review.

The systematic search led to the identification of 24 articles from three databases, of which 12 were based on animal studies and 12 on humans. A flowchart of the final selection of items is shown in Figure 2. Main characteristics of the selected articles are summarized in Table 1.

### 3.4. Discussion

#### 3.4.1. Animal Studies

Among twelve studies based on animal models, four studies tested diet supplementation with soy [74], dapaglifozin [75], indole-3-propionic acid [76], and hesperidin [77]. Three studies focused on fecal transplantation [78,79,80], and two on antibiotic treatment [81,82]. Other studies investigated the SCFA receptor [29] and germ-free mice [83]. Only three studies reported data on BP levels [29,49,77]. Nine studies analyzed arterial stiffness by recording PWV by aortic doppler (see Table 1).

**Table 1:** Characteristics of animal studies.

Authors	*n*	Marker of VA	Intervention	Duration	Effect on Vascular Ageing	Mechanisms Linked to Microbiota
Guirro, M., 2020 [77]	48	Neuraminidase circulating levels	Hesperidin treatment; two diets for 9 wk (*n* = 24): standard diet and cafeteria (CAF) diet	9 weeks of diet + 8 weeks of hesperidin	CAF feeding resulted in increased endothelial dysfunction, arterial stiffness, and inflammation. Hesperidin supplementation reduced SBP and markers of arterial stiffness in CAF-fed rats	Urinary metabolites of hesperidin were positively correlated with Bacteroidaceae family.
Liu, H., 2020 [78]	35	PWV at the left common carotid artery	Gavage with feces from either healthy donors (controls) or myocardial infarction patients (CAD) + high fat diet	12 weeks	Mice treated with CAD feces had higher vascular stiffness than controls (Controls: 2.75 ± 0.29 m/s vs. CAD: 3.59 ± 0.27 m/s; *p* = 0.043). No BP data.	In mice treated with CAD feces: increased LPS and pro-inflammatory cytokines; increased activated TH17 cells; reduced Treg cells.
Battson, M.L., 2019 [79]	40	AorticPWV (doppler)	Fecal transplantation, 10 controls and 10 obese mice received healthy microbiota, and 10 and 10 received obese microbiota	8 weeks	Control mice receiving microbiota of obese subjects had higher PWV. *Akkermansia* abundance inversely related to PWV. No BP data.	Obese mice had reduced *Clostridia* and *Oscillospira*. Control mice and obese mice receiving microbiota of obese subjects had higher level of Bacteroides sp. Control mice receiving microbiota of obese subjects had reduced level of *Akkermansia*.
Natarajan, N., 2016 [29]	10	Aortic stiffness (PWV by doppler and ex vivo)	Gpr41 KO group vs Grp41 WT group	3 and 6 months	At 6 months PWV was significantly higher in KO mice vs WT mice, with similar compliance in ex vivo analysis, suggesting functional vascular alteration. KO mice presented isolated systolic hypertension at baseline	Gpr41 (SCFA receptor) localizes in the vascular endothelium. Vascular endothelium is essential for SCFA-mediated vasodilation to occur, as vasodilation is absent in endothelium-denuded vessels ex vivo.
Edwards, J.M., 2020 [83]	12	Resistance arteries stiffness (ex vivo)	Ex vivo evaluation of vascular stiffness		Resistance arteries from male GF mice present increased vascular stiffness. No changes in vascular stiffness in arteries from female mice. No BP data.	Microbiota influenced the vasoconstriction response.
Cross, T.W.L., 2017 [74]	40	Aortic PWV (doppler)	Ovariectomy vs sham surgery; soy-rich vs soy-free diet	28 weeks	PWV was lowered with soy feeding but was not affected by ovariectomy. No BP data.	Soy-rich diet modified intestinal microbiota composition (lower F:B ratio).
Battson, M.L., 2018 [81]	36	Aortic PWV (doppler)	Standard diet (SD) (*n* 12) or Western diet (WD) (*n* 24) for 5 months, then WD mice were randomized to receive broad-spectrum antibiotic cocktail (WD + Abx) or placebo (*n* 12/group) for 2 months	7 months	PWV progressively increased in WD mice during the 7-month intervention. In WD + Abx, PWV was completely normalized to SD levels. No BP data.	WD had increased Firmicutes and decreased Bacteroidetes and Actinobacteria. Abundance of numerous bacterial taxa were altered by diet; in particular, *Bifidobacterium* spp. were significantly more abundant in SD animals compared with WD.
Brunt, V.E., 2019 [82]	73	Aortic PWV (doppler); ex vivo intrinsic mechanical stiffness	Cocktail of broad-spectrum, poorly absorbed antibiotics in drinking water vs placebo. 4 groups: young controls (YC); young antibiotics (YA); old controls (OC); old antibiotics (OA).	3–4 weeks	At baseline, PWV was higher in OC and OA vs YC (*p* < 0.01). PWV increased in YC but not in YA during intervention. In OA, PWV was reduced at the end of the intervention. Antibiotic treatment in old mice was associated with a partial improvement back towards young levels (*p* = 0.047 vs. OC). Aortic elastin protein expression was lower in OC vs. YC (*p* = 0.02), but was restored in OA. No BP modifications were registered.	Ageing was associated with greater alpha diversity. Old mice demonstrated several bacterial markers of gut dysbiosis and/or inflammation. Three-fold age-related increase in circulating plasma TMAO levels. In both young and old mice, antibiotic treatment suppressed TMAO levels.
Lee, D.M., 2018 [75]	47	Aortic PWV (doppler); ex vivo	(1) standard diet; (2) standard diet + dapagliflozin (60 mg dapagliflozin/kg diet). Controls (*n* = 11); Controls + dapa (*n* = 12); Diabetics (Db) (*n* = 12); Db + dapa (*n* = 12).	8 weeks	Dapagliflozin treatment improved both endothelium-dependent dilatation (EDD) and Endothelium-independent dilation (EID) in Db mice. PWV was negatively and EID-EDD positively correlated with *Akkermansia* abundance. PWV was positively correlated with Firmicutes and F:B ratio. No BP data.	Significantly reduced richness and diversity in the Db + dapa group compared to controls. Bacteroidetes and Proteobacteria were influenced by dapagliflozin treatment in Db + dapa. Db + dapa had a significantly lower F:B ratio than the other treatment groups. *Oscillospira* was significantly reduced in the Db + dapa compared to all other groups
Lee, D.M., 2020 [76]	48	Aortic PWV (doppler)	Standard (SD) vs Western diet (WD). Indole-3-propionic acid (IPA) vs placebo. (1) SD + placebo, (2) WD +placebo, (3) SD + IPA, 4) WD + IPA. (*n* = 12 mice/group).	5 months	IPA supplementation did not affect PWV in WD, but impaired PWV in SD. *Bifidobacterium* reduction by WD was related to PWV. No BP data.	WD feeding decreased *Bifidobacterium*. Reduced abundance of *Bifidobacterium* was observed in SD + IPA.
Trikha, S.R.J., 2021 [80]	10	Aortic PWV (doppler)	2 age-matched male and 2 female (1 of each lean [LM], and 1 obese [OBM]) microbiota donors to form cohorts 1 and 2 of inoculated mice.		PWV was increased in OBM mice vs. GF mice. In cohort 2, OBM mice displayed a marked increase in PWV vs. LM mice. No BP data.	Mouse microbiota profiles clustered according to their transplant donor groups. Taxa appear to be driving this separation, *Bacteroides ovatus* and *Parabacteroides diastonis* were consistently associated with LM mice.

VA stands for Vascular ageing; SBP, systolic blood pressure; PWV, pulse wave velocity; LPS, lipopolysaccharides; Gpr, G-protein coupled receptor; SCFA, short-chain fatty acid; GF, germ-free; F:B, Firmicutes/Bacteroidetes ratio; TMAO, trimethylamine-N-oxide.

Supplementation studies. In all studies, independent of the type of supplement, the modifications of gut microbiota were associated with parallel modification to arterial stiffness. In particular, it seems that the changes of the gut microbiota linked to a better configuration are correlated with lower arterial stiffness. (i) In rats selectively bred for low running capacity, soy supplementation significantly improved their blood lipid profile, adipose tissue inflammation, and aortic stiffness; it shifted the cecal microbiota toward a lower F:B ratio. Soy-fed rats had lower mRNA expression of CD11c (inflammatory macrophage marker) and of the proinflammatory cytokine IL-6 [74]. (ii) Diabetic mice presented higher cfPWV than controls. Supplementation with dapaglifozin was associated with reduced microbiota diversity and richness, but failed to improve arterial stiffness in the study by Lee DM. Interestingly, arterial stiffness was negatively associated with *Akkermansia* abundance and positively with F:B ratio [75]. (iii) The supplementation with indole-3-propionic acid (a microbial metabolite of the essential aromatic amino acid, tryptophan) did not improve cfPWV in mice fed a western diet, and even worsened cfPWV in control mice, which also presented a reduced abundance of *Bifidobacterium* [76]. (iv) Supplementation with hesperidin resulted in higher urinary excretions of hippurate and other polyphenols metabolites. As most polyphenols are metabolized by gut microbiota before being absorbed, in this study, urinary metabolites of hesperidin were positively correlated with a microbial family, the Bacteroidaceae (phylum Bacteroidetes). From the vascular point of view, hesperidin supplementation was able to reduce both the circulating levels of neuraminidase (a biological marker of arterial stiffness [84]) and the systolic BP [77]. These results show a biological effect of polyphenols on vascular ageing, and indicate gut microbiota modification as the potential mechanism of the effect.

Fecal transplantation. Arterial stiffness was associated with gut microbiota modifications induced by fecal transplantation. In particular, PWV was positively associated with Clostridium genus, which contains most of the deleterious *Clostridium* species (e.g., *C. botulinum*, *C. perfringens*, *C. difficile*), and with gut permeability and obese microbiota, whereas it was negatively associated with Akkermansia abundance. (i) Local carotid stiffness was investigated in mice fed with a high-fat diet and gavaged with gut microbiota of either healthy donors (“Con” group) or patients with myocardial infarction (“CAD” group). The characteristics of the gut microbiota from CAD patients were transmissible and associated with low fermentation and high inflammation, and with increased abundance of *Clostridium symbiosum* (Clostridium genus) and Eggerthella genus. CAD mice also presented a higher carotid stiffness than the Con group of about 1 m/s [78]. (ii) An interesting study from Battson M et al. investigated fecal transplantation in four groups: control mice fed with either normal microbiota (Con + Con) or microbiota from obese mice (Con + Ob); and obese mice fed with either normal microbiota (Ob + Con) or obese (Ob + Ob) microbiota. Higher PWV was observed after obese microbiota gavage in control mice, together with altered gut permeability and SCFA content. Importantly, Akkermansia abundance was strongly inversely related to PWV with r = −0.8 (*p* < 0.0001) [79]. (iii) In another experimental study, the microbiota from either lean (LM) or obese (OBM) patients was used to feed two cohorts of mice: one from male and one from female patients. Aortic stiffness was higher in OBM than germ-free mice, and in cohort 2, it was also higher in OBM than LM mice. Mouse microbiota profiles clustered according to their transplant donor groups, possibly explaining the difference in arterial stiffness [80].

Antibiotic treatment. In both studies that we found, antibiotic treatment induced deep changes in gut microbiota composition, which were associated with parallel changes in arterial stiffness. (i) The role of antibiotic treatment was investigated in mice fed with a western diet for 5 months. During the study, mice presented a progressive increase in aortic PWV, which was completely reversed by 2-month antibiotic supplementation. The western diet increased the F:B ratio and Ruminococcus abundance; it reduced the abundance of Bifidobacterium, and increased inflammatory markers like LPS-binding protein, IL-6, plasminogen activator inhibitor-1, which were normalized after the antibiotic treatment. Four groups were analyzed: old control mice (OC), old mice fed with antibiotic supplementation (OA), young control mice (YC); young mice with antibiotics (YA). Old mice presented several markers of dysbiosis and inflammation, with higher levels of TMAO and PWV. During the intervention, PWV increased in young mice without antibiotic supplementation only. In old mice, antibiotic treatment was associated with: (i) partial PWV improvement (*p* = 0.047 vs. OC); (ii) increased aortic elastin expression; (iii) suppressed TMAO levels [81]. (ii) In one study from Brunt V et al., 35 young and 38 old mice were treated with antibiotic supplement for 3 to 4 weeks. After the supplementation, most of major phyla were suppressed. The intervention restored arterial stiffness in old mice to normal levels, and normalized oxidative stress and inflammation [82].

The role of SCFA receptor Gpr41 in vascular function was investigated in Gpr41-KO mice. Gpr41 was found in the vascular endothelium, which was necessary for SCFA-mediated vasodilation. At baseline, Gpr41-/- mice presented isolated systolic hypertension, but with no differences in plasma renin concentration between WT and KO mice. At 6mo, Gpr41-/- mice showed accelerated vascular ageing with higher PWV than wild mice. Of note, ex vivo analysis at 6mo showed no difference in tensile vessel properties. The disparity between higher PWV and unchanged structural vessel properties indicates that functional alterations may occur before structural modifications exist [29].

The gut microbiota influences the resistance properties of arteries, with increased stiffness in male germ-free mice with respect to either wild or female germ-free mice [83].

All these experimental animal studies consistently show a strict correlation between modification of gut microbiota and arterial stiffness. Moreover, some of them highlight the role of inflammation as a mediator between the gut microbiota and arterial stiffness.

#### 3.4.2. Human Studies

Among studies on humans (see Table 2), we found five cross-sectional and seven intervention studies. Ten studies present information on BP, and six employed cfPWV as a measure of arterial stiffness. Three studies did not directly evaluate modification in microbiota.

Cross-sectional studies. In this study setting, a correlation between gut microbiota modifications and arterial stiffness parameters was consistently found across the included studies. In particular, the abundance of beneficial bacteria (mainly butyrate producers) is constantly associated with lower arterial stiffness. (i) Menni et al. investigated more than 600 women from the TwinsUK registry, and measured tonometric carotid-femoral PWV and microbiota composition. They found that gut microbiome diversity was significantly inversely associated with arterial stiffness. PWV was also negatively associated with the abundance of Ruminococcaceae family bacteria, which are beneficial butyrate-producing bacteria linked to lower endotoxemia [85]. (ii) In 10 hemodialysis patients, Firmicutes and Bacteroidetes phyla were the most abundant. Faecalibacterium spp. (butyrate producer from the Oscillospiraceae family, class Clostridia, phylum Firmicutes) were positively associated with total carbohydrate intake (ρ = 0.636; *p* = 0.048) and negatively associated with cfPWV (ρ = −0.867, *p* = 0.001). Lipopolysaccharide-Binding Protein, a marker of bacterial translocation through the intestinal barrier and endotoxemia, was negatively associated with butyrate-producing bacteria [86]. This result supports the association between the favorable microbiota composition and vascular ageing, through a reduction of the systemic inflammation derived from leaky gut syndrome. (iii) In sixty-nine subjects not treated for hypertension who underwent an ambulatory BP monitoring, an ambulatory arterial stiffness index (AASI) was obtained. AASI is calculated from the regression line between 24 h systolic and diastolic BP values, and is believed to be a marker of arterial stiffness. Although no definite evidence exists on its accuracy, at least it seems able to predict cardiovascular events. No association was found between microbiota diversity indexes and AASI. AASI was associated with lower abundance of Lactobacillus spp. and higher abundance of several deleterious species from the genus Clostridium [87]. (iv) In children with chronic kidney disease with different categories of estimated glomerular filtration rate (G1: eGFR ≥ 90 mL/min/1.73 m^2^, 9.5 years-old; G2-G3: eGFR 30–89, 13.7 ys), carotid-PWV was correlated to the severity of the disease. Although various beneficial bacteria (Lactobacillus, Bifidobacterium, Akkermansia) were not influenced by the severity of the disease, genus Lactobacillus abundance was negatively correlated with PWV [88].

Supplement intervention. Three studies investigated the effect of supplementation on gut microbiota and arterial stiffness, without any significant result. Only one study found a significant effect on arterial stiffness reduction associated with a specific microbiota pattern, but the effect was only marginal. (i) A total of sixty-six healthy men were enrolled in a randomized, double-blind placebo-controlled trial where two forms of aronia supplementation were compared to a placebo after 12-week treatment: a (poly)phenol-rich aronia extract, and an aronia fruit powder. No effect on aortic stiffness was registered. Gut microbial diversity was very high and did not show significant variation among the treatment groups after aronia intake; the aronia extract group presented an increased abundance of genus Anaerostipes (butyrate producer, family Lachnospiraceae, class Clostridia) [89]. (ii) A relatively young uncontrolled hypertensive sample was randomized to receive garlic (*n* 23) supplementation or a placebo (*n* 26) for 12 weeks. Tonometric cfPWV presented a trend for reduction in the garlic group (from 12.8 to 12.1 m/s), but with no statistical difference versus the placebo. Of note, SBP was reduced in garlic versus the placebo arm, with a mean difference of 10 mmHg. The garlic group presented an increase in Lactobacillus and Clostridium spp, without deeper characterization of the bacterial species [90]. (iii) A total of twelve young men were studied after consumption of 2 eggs/day for 2 weeks in a non-randomized trial. Brachial-ankle PWV and endothelial function improved, with no effect on BP, inflammation, oxidative stress, or TMAO. Microbiota was not modified, but a reduction of tryptophan degradation was observed [91].

Exercise intervention. Both the following studies found a significant relationship between the gut microbiota and arterial stiffness surrogates. In particular, the second study, focused on a very intense exercise training program, found a significant microbiota modification after the training, associated with improvement of the augmentation index (an indirect index of arterial stiffness, which is associated both with vascular peripheral resistances and with the phenomenon of the pulse wave reflection). Unfortunately, the trial was not randomized and the augmentation index is not only related with arterial stiffness, but also to the peripheral resistances and heart rate. This mines the interpretation of the results. (i) A crossover trial with 5 wks of exercise training and 5 wks of washout period found a significant positive correlation between Clostridium difficile and arterial stiffness in 33 men, measured by the cardio-ankle vascular index (CAVI), (r = 0.306, *p* = 0.016) and SBP, but with no exercise effect. Microbiota diversity was not affected by exercise, but the relative abundances of Oscillospira and Clostridium difficile were increased and decreased by exercise, respectively [92]. Considering that Oscillospira species (family Oscillospiraceae, class Clostridia) are associated with leanness and may have anti-inflammatory properties [93], the results of this study support the beneficial role of exercise training in microbiota composition. (ii) In a non-randomized interventional trial, 24 obese adolescents underwent a 6-wk program of endurance/strength training for 5 h/day and 6 d/wk, together with caloric restriction. The subendocardial viability ratio, an index of the workload of the left ventricle depending on ventricle-vascular coupling, and the augmentation index were improved by the program, with no significant change in BP. Microbiota diversity increased, together with the abundance of the Christensenellaceae family, which is inversely related to the host body mass index [94].

Studies without direct microbiota measures. Three studies present results on arterial stiffness modification in relation to either comorbidities or supplementation design, assuming an indirect role of gut microbiota. (i) In the paper by Ponziani et al., 39 patients with suspected small intestinal bacterial overgrowth (SIBO) were included. Vitamin-K2 status was measured, and Vitamin-K2 intake and carotid PWV were obtained. In patients with confirmed SIBO (*n* = 12), despite similar dietary vitamin-K2 intake, measured vitamin-K2 status was markedly reduced versus the no-SIBO group (*p* = 0.02), suggesting an altered vitamin-K2 production by intestinal bacteria. Median PWV was significantly higher in the SIBO group than the no-SIBO group (10.25 m/s vs. 7.68 m/s; *p* = 0.002). Furthermore, in both groups, vitamin-K2 status was significantly correlated with carotid PWV (R2 = 0.29, *p* < 0.001) [95]. This study supports the role of gut dysbiosis in vascular ageing. (ii) In a randomized trial, 15 patients received 1-month cocoa extract with 130 mg epicatechin and 560 mg procyanidins (group D1-10), 15 patients received 20 mg epicatechin and 540 procyanidins (group D2-10), and were compared with the placebo (*n* = 15). Of note, both epicatechin and procyanidins are catabolized by gut microbes in the colon. CfPWV was reduced in the D1-10 group by about 1 m/s versus the placebo and by 0.8 m/s versus D2-10 group. SBP was also reduced in D1-10 versus the control and D2-10 groups. In this study, the reduction of SBP could explain the observed variation in PWV. The impact of cocoa flavonols on vascular health was mainly linked to epicatechin, and is mediated by epicatechin metabolites, which in turn depends on gut microbiota metabolism [96]. (iii) One study focused on the effect of equol, a microbial-derived metabolite of the isoflavone daidzein. Equol is produced by gut microbiota after soy intake in almost one-third of the Western population. Acute soy supplementation slightly reduced cfPWV only in equol producers (−0.2 m/s) at 24 h, with no change in BP [97].

Even if a direct association with microbiota composition and function has not been addressed, the three studies presented here show a significant correlation between vascular ageing and the metabolites of gut microbiota. This confirms the role of gut microbiota in modulating human systemic biological pathways.

Antibiotics in humans. In contrast to what has been shown in animal studies, this paragraph aims to warn the reader not to consider antibiotic treatment beneficial for cardiovascular health in humans. Indeed, the role of antibiotics in humans is not fully established. Studies investigating the role of antibiotics in microbiota and the effect on cardiovascular risk are lacking. According to the literature, it appears that antibiotics impact the gut microbiota, reducing bacterial diversity and changing relative abundances [98]. They were also found to enhance pathways linked to increased atherosclerosis [99]. Long-term use of antibiotics in late adulthood has been associated with all-cause and cardiovascular mortality [100]. Macrolide antibiotic consumption is associated with increased risk for sudden cardiac death or ventricular tachyarrhythmias and cardiovascular death, but not increased all-cause mortality [101]. Furthermore, no association with long-term cardiovascular risk (ranging from >30 days to >3 years) was noted in observational studies or randomized controlled trials on treatment with macrolides [102]. A significant association was found between fluoroquinolone use and an increased risk for arrhythmia and cardiovascular mortality [103]. Antibiotic exposure in infancy was associated with a slightly increased risk of childhood overweight and obesity [104]. The pooled colorectal cancer risk was increased among individuals who ever used antibiotics, particularly for broad-spectrum antibiotics [105]. The pooled breast cancer risk was modestly increased among individuals who ever used antibiotics [106].

Limitations. This study presents several limitations. Firstly, due to the small number of studies and great heterogenicity among them, it was not possible to make a meta-analysis of the results. Second, the quality of most of the studies was questionable, due either to sample size, or to the assessment of microbiota or arterial stiffness. Third, for the reason just mentioned, it was not possible to entirely follow the PRISMA statement for systematic review.

### 3.5. Conclusions

From the available literature, we found an association between gut microbiota composition and arterial stiffness. We identified two association patterns, consistently present in most animal and human studies: (i) a direct correlation between arterial stiffness and abundances of bacteria associated with altered gut permeability and inflammation (mainly from the *Clostridium* genus), as well as with biological markers of inflammation; (ii) an inverse relationship between arterial stiffness, microbiota diversity, and abundances of bacteria associated with most fit microbiota composition (butyrate producers, *Akkermansia*, *Bifidobacterium*, *Ruminococcaceae*, *Faecalibacterium*, *Lactobacillus*).

While in animal studies most of the interventions were able to show a stable link between microbiota modification and arterial stiffness, in humans that was not the case. In particular, none of the identified interventional trials was able to demonstrate this relationship. However, most strikingly, nearly half of human studies measured BP, and very few adjusted the vascular analyses for BP variation, which is a major determinant of arterial stiffness.

The main finding of this review is the lack of large randomized interventional trials in humans that test the role of gut microbiota modifications on arterial stiffness, and take into account BP and hemodynamic alterations.

**Table 2:** Characteristics of human studies.

Authors	*n*	Marker of VA	Intervention	Duration	Effect on Vascular Ageing	Mechanisms Linked to Microbiota
Rodriguez-Mateos, A., 2018 [96]	45	cfPWV	DP1-10 group: cocoa extract with 690 mg (130 mg epicatechin; 560 mg DP2-10 procyanidins). DP2-10 group: cocoa extract with 560 mg (20 mg epicatechin; 540 mg DP2-10 procyanidins). Controls.	1 month	DP1-10 group: decrease in PWV at 1 mo of −1.0 m/s (95% CI: −1.6, −0.4 m/s) compared with the control and of −0.8 m/s (95% CI: −1.4, −0.2 m/s) compared with DP2-10. Decrease in SBP at 1 month in both treatment groups.	Epicatechin is absorbed via the colon after catabolism by the microbiota; Pro-cyanidins are also subject to microbiome-mediated catabolism.
Istas, G., 2019 [89]	66	cfPWV; AIx	Aronia whole fruit capsule: 12 mg (poly)phenols; aronia extract capsule: 116 mg (poly)phenols.	Acute: 0–2 h Chronic: 0–12 weeks	No significant difference in PWV and BP.	The aronia extract group: higher abundance of *Anaerostipes*; the aronia whole fruit group: increases in Bacteroides.
Taniguchi, H., 2018 [92]	33	CAVI	Exercise program (*n* = 16) and control period (*n* = 17).	10 weeks	Changes in *Clostridium Difficile* were positively correlated both with CAVI (r 0.31, *p* 0.02; no effect of exercise) and with SBP	Diversity and composition of microbiota were not affected by exercise; exercise increased the relative abundance of *Oscillospira* and decreased the abundance of *C. Difficile*
Menni, C., 2018 [85]	617	cfPWV	Observational study in female twins.	N/a	Carotid-femoral PWV is inversely correlated with gut microbiome diversity and with the abundance of specific microbes in the gut (Ruminococcaceae family bacteria). Analysis was adjusted for MAP.	N/a
Biruete, A., 2019 [86]	10	cfPWV	Observational study in hemodialysis patients.	N/a	*Faecalibacterium* spp. (with anti-inflammatory properties), was negatively associated with aortic PWV. F:B ratio was positively associated with SBP.	N/a
Ponziani, F.R., 2017 [95]	39	Carotid PWV	Patients with small intestinal bacterial overgrowth (SIBO).	N/a	PWV was increased in the SIBO group compared to the no-SIBO group (10.25 m/s vs 7.68 m/s; *p* = 0.002). dp-ucMGP levels (marker of low vitamin-K2 status) correlated with PWV in whole population. No BP data.	Dietary vitamin-K2 intake does not correlate with vitamin-K2 status (measured by dp-ucMGP serum levels). The gut microbiota is crucial for overcoming dietary vitamin-K2 insufficiencies.
Ried, K., 2018 [90]	49	cfPWV (tonometry)	Kyolic Aged Garlic Extract vs placebo.	12 weeks	No significant differences in PWV between groups and intra-group before and after treatment. Garlic reduced SBP.	Increase of *Lactobacillus* and *Clostridia* species in the garlic group. *Faecalibacterium prausnitzii* markedly increased in the placebo group.
Hazim, S., 2016 [97]	28	cfPWV	Soy isoflavones acute supplementation.	3 days	Acute soy intakes modified cfPWV only in equol producer subjects at 24 h; equol concentrations were significantly correlated with changes in cfPWV. No changes in BP.	N/a
Huang, J., 2020 [94]	24	AIx75; SEVR	Obese individuals underwent exercise: endurance/strength training 5 h/day, 6 days/week; diet: calorie-restricted.	6 weeks	Significant increase of SEVR; reduction of AIx. No changes in BP.	Increase in intestinal microbial diversity; abundance of *Lactobacillales*, *Bacilli*, *Streptococcaceae*, and *Veillonella* were significantly reduced. Christensenellaceae were significantly enhanced; changes in *Cronobacter*, *Lachnospiraceae* UCG-003, and *Helicobacter* were all positively or negatively associated with the changes in SEVR, AIx75.
Dinakis, E., 2021 [87]	69	AASI	Observational study.		No associations were found between alpha diversity and AASI; no significant clustering patterns of AASI; Small but positive correlation between plasma butyrate levels and AASI. No BP data.	AASI was associated with lower abundance of *Lactobacillus* spp. and higher abundance of several species from the genus *Clostridium.*
Liu, X., 2022 [91]	12	baPWV/FMD	2 eggs/day in healthy young men.	2 weeks	Egg consumption improved baPWV and FMD. No effect on inflammation and oxidative stress. No changes in BP.	No change in taxonomy, alpha and beta diversity; reduced tryptophan degradation.
Hsu, C.N., 2018 [88]	86	carotid-PWV (echo-tracking)	Observational study on children and adolescents with chronic kidney disease (CKD).	N/a	Carotid-PWV was elevated in children with CKD and eGFR category G2–G3 compared to those with eGFR category G1. 65% of children and adolescents with CKD G1–G3 had BP abnormalities on ABPM.	CKD children with an abnormal ABPM profile had lower abundance of the genus *Prevotella*; the abundances of genera *Bifidobacterium* and *Lactobacillus* were correlated with urinary TMAO level.

VA stands for Vascular ageing; SBP, systolic blood pressure; cfPWV, carotid-femoral pulse wave velocity; CAVI, cardio-ankle vascular index; MAP, mean arterial pressure; AIx75, augmentation index corrected for heart rate at 75 bpm; SEVR, sub-endocardial viability ratio; AASI, ambulatory arterial stiffness index; baPWV, brachial-ankle pulse wave velocity; FMD, flow-mediated dilation; eGFR, estimated glomerular filtration rate; eGFR categories: G1 ≥ 90 mL/min/1.73 m^2^, G2 50–89, G3 30–59; ABPM, ambulatory blood pressure measurement; TMAO, trimethylamine-N-oxide.

## Data Availability

No new data were created or analyzed in this study. Data sharing is not applicable to this article.
